# Facile Synthesis of Mesoporous NiCo_2_O_4_ Nanosheets on Carbon Fibers Cloth as Advanced Electrodes for Asymmetric Supercapacitors

**DOI:** 10.3390/nano15010029

**Published:** 2024-12-27

**Authors:** Xiang Zhang

**Affiliations:** Department of Energy and Power Engineering, North University of China, Taiyuan 038507, China; zhangxiang@bit.edu.cn

**Keywords:** asymmetric supercapacitors, carbon fibers, mesoporous nanosheets

## Abstract

The NiCo_2_O_4_ Nanosheets@Carbon fibers composites have been successfully synthesized by a facile co-electrodeposition process. The mesoporous NiCo_2_O_4_ nanosheets aligned vertically on the surface of carbon fibers and crosslinked with each other, producing loosely porous nanostructures. These hybrid composite electrodes exhibit high specific capacitance in a three-electrode cell. The asymmetric supercapacitor (NiCo_2_O_4_ Nanosheets@Carbon fibers//Graphene oxide) displayed a high specific capacitance of 91 F g^−1^ and excellent cycling stability with a capacitance retention of 94.5% at 5 A g^−1^ after 10,000 cycles. The device also achieved a notable energy density of 52 Wh kg^−1^ coupled with a power density of 3.5 kW kg^−1^ and a high power density of 7.1 kW kg^−1^ with an energy density of 21 Wh kg^−1^. This study shed light on the great potential of this asymmetric device as future supercapacitor.

## 1. Introduction

With the growing popularization of portable electronics and electronic devices, energy storage devices with high performance are in demand [1,2,3,4]. Supercapacitors have drawn considerable research attention due to the advantages of high power density, fast charge–discharge time, being environmentally benign, low cost, and good cycling stability [5,6]. However, they are limited in practical application by the low energy density feature, which depends on the cell capacitance (C) and potential window (V) in the form of E = CV^2^/2 [7,8,9]. A promising way to overcome this drawback is to develop asymmetric supercapacitors with a battery type faradic electrode as an energy source and a capacitive electrode as a power source, where the operating voltage can be increased [10,11]. Another route is to improve the capacitance of the electrode materials. Among various materials for supercapacitor electrodes, spinel NiCo_2_O_4_, a low cost binary metal oxide, has drawn worldwide attention as an ideal pseudocapacitive electrode material due to the existence of Ni^2+^/Ni^3+^ and Co^2+^/Co^3+^ redox couples [12,13,14,15,16]. With a pity, the electrical conductivity of NiCo_2_O_4_ is poor, limiting its utilization as supercapacitor materials. Combining these poor conductive metal oxides with conductive carbon materials is an effective way to produce high-performance hybrid materials [17,18,19,20]. Among various carbon materials, one-dimensional carbon fibers (CFs) have received extensive attention as a class of chemically stable supports to enhance the electrical conductivity of metallic oxides as the efficient electrode materials because of their one-dimensional properties, high specific surface area, and good electrical conductivities. On account of their excellent mechanical property and easy preparation, it has been considered as the promising conductive support for metal oxides [21,22].

In this report, we fabricated a NiCo_2_O_4_ Nanosheets@Carbon fibers cloth composite and paired it with reduced Graphene oxide (rGO) to form an asymmetric supercapacitor. This asymmetric device delivered a high energy density and power density, and excellent cycling stability.

## 2. Experimental

### 2.1. Materials

Nickel (ll) nitrate hexahydrate Ni(NO_3_)_2_·6H_2_O with 99.0% assay from Sigma-Aldrich (St. Louis, MO, USA) and cobalt (II) nitrate hexahydrate Co(NO_3_)_2_·6H_2_O with 99.0% assay from Sigma-Aldrich were employed as precursors without further modification. Carbon fibers cloth was obtained from Toho Tenax Co. Ltd., Tokyo, Japan. All chemicals used were of analytical grade and were directly utilized without undergoing further purification. Aqueous solutions were prepared using deionized water with a conductivity of 18.2 MΩ·cm by the Elga DI water system.

### 2.2. Synthesis of NiCo_2_O_4_ Nanosheets@Carbon Fibers Cloth by Co-Electrodeposition

Firstly, carbon fibers (CFs) were cleaned using ultrasound in deionized water and ethanol for 30 min, followed by drying in an oven. Subsequently, the CF underwent a heat treatment in a tube furnace at 240 °C for 3 h in air, with a heating rate of 5 °C·min^−1^, to enhance structural stability. Finally, the CF was subjected to ultrasonic cleaning in 2 M HCl to remove impurities, followed by washing with deionized water and overnight drying at 80 °C. The resultant carbon fibers cloth (CF) was used as the substrate for the growth of NiCo_2_O_4_ nanosheets.

The NiCo_2_O_4_ NS@CF was prepared through a facile co-electrodeposition method followed by calcination. The co-electrodeposition process was carried out in a three-electrode system using a mixed precursor solution of Ni(NO_3_)_2_·6H_2_O and Co(NO_3_)_2_·6H_2_O with a molar ratio of 1:2. A configuration size of 2 cm × 2 cm carbon fiber (CF) was used as the working electrode, while a platinum foil (Pt) and a Ag/AgCl were utilized as the counter and reference electrode, respectively. The electrodeposition potential was 1.5 V at room temperature for 10 min. Subsequently, the as-deposited CF was rinsed with deionized water and ethanol, dried in a vacuum oven at 60 °C overnight, and calcined at 300 ◦C for 2 h in air with a heating rate of 1 °C min^−1^. The mass loading of NiCo_2_O_4_ nanosheets on carbon fibers cloth was 1 mg/cm^2^.

### 2.3. Characterizations

The prepared samples were characterized through various physicochemical techniques. The crystal structure was analyzed using X-ray diffraction (XRD) on a Bruker D8 diffractometer. The more detailed elemental composition and the oxidation state of the NiCo_2_O_4_ NS@CF are carried out by X-ray photoelectron spectroscopy (XPS, AXIS Ultra). Scanning electron microscopy (SEM, Zeiss, 5.0–20.0 kV) was utilized to investigate the morphologies and element distribution of the samples.

### 2.4. Electrochemical Performance Test

The cyclic voltammetry measurement was conducted in a potential range between −0.2 and 0.55 V at different scan rates ranging from 5 to 100 mV s^−1^. The galvanostatic charge–discharge processes were performed at different current densities in 1 M KOH aqueous electrolyte. The cyclic stability was evaluated by the current density of 5 A g^−1^ for 5000 cycles, which were acquired using an Arbin testing system (MSTAT).

## 3. Results and Discussion

**Figure 1:**
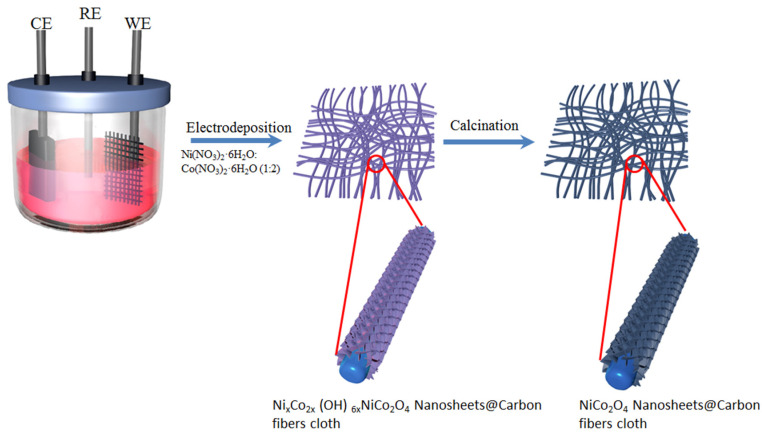
Schematic representation of the procedure used to fabricate NiCo_2_O_4_ Nanosheets@Carbon fibers cloth.

Our synthesis strategy consists of two steps, as illustrated in Figure 1: Firstly, the process involves co-electrodeposition of a bimetallic (Ni, Co) hydroxide precursor onto a carbon fiber cloth. Secondly, the resulting bimetallic (Ni, Co) hydroxides undergoes calcination to form nickel cobaltite. The electrolyte utilized in this process contains Ni^2+^, Co^2+^, and NO_3_^−^ ions, maintaining a molar ratio of Ni^2+^ to Co^2+^ at 1:2. As an electric current passes through the electrolyte, the reduction of NO_3_^−^ ions occurs at the cathode, producing OH^−^ ions.

The presence of OH^−^ ions increases the local pH value, facilitating the uniform precipitation of mixed metal (Ni, Co) hydroxide on the surface of the carbon fibers. The whole process involves an electrochemical reaction followed by the subsequent precipitation of mixed metal hydroxide, as elucidated by the following two equations:

NO_3_^−^ + 7H_2_O + 8e^−^ → NH_4_^+^ + 10 OH^−^ (1)

xNi^2+^ + 2x Co^2+^ + 6x OH^−^ → Ni_x_Co_2x_ (OH)_6x_ (2)

Subsequently, the metal hydroxides are subjected to high-temperature calcination in air to produce desired nickel cobaltite product on the carbon fibers cloth. This transformation is described by the oxidation reaction as follows:

Ni_x_Co_2x_ (OH)_6x_ + 0.5x O_2_ → x NiCo_2_O_4_ + 3x H_2_O (3)

**Figure 2:**
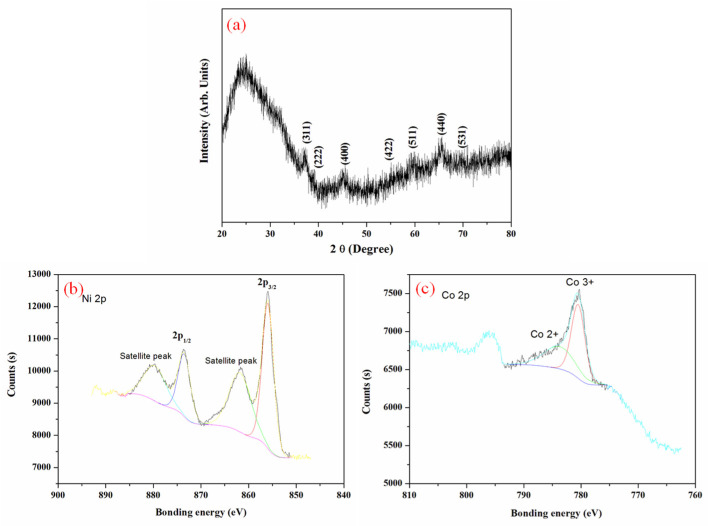
(**a**) X-ray diffraction patterns and (**b**,**c**) XPS of NiCo_2_O_4_ Nanosheets@Carbon fibers.

The crystal structural and phase characterizations were conducted using XRD and XPS studies. A broad peak at 24.9°, a typical peak of (002) plane of graphitic carbon, was observed and assigned to the diffraction patterns of the CF in Figure 2a and Figure S8. The other diffraction peaks at 2θ values of 18.9°, 36.7°, 38.4°, 44.6°, 55.4°, 59.1°, 65.0°, and 68.3° matched well to the (111), (311), (222), (400), (422), (511), (440), and (531) of spinel NiCo_2_O_4_ (JCPDF card no. 20-0781) in Figure 2a and Figure S9. No other peaks of nickel or cobalt compounds were observed, indicating that the synthesized nanosheets were only NiCo_2_O_4_. XPS spectra were measured and shown in Figure 2b,c. The Ni 2p spectrum was composed of two well-defined single peaks and two shakeup satellite peaks. The peaks at 856.6 and 874.6 eV were assigned to Ni 2p_3/2_ and Ni 2p_1/2_, respectively, whereas the peaks at 861.4 and 882.1 eV were two satellite peaks [18,23,24]. The Co 2p spectrum comprised one spin-orbit doublet characteristics and one shakeup satellite peak. The main peaks around 780.4 eV belonged to Co^3+^, while the weak peaks at 781.85 eV corresponded to Co^2+^ [18,25]. The satellite peak was seated at approximately 796.2 eV. It indicated that the NiCo_2_O_4_ NS@CF composite contained Ni^2+^ and the electron couple of Co^3+^/Co^2+^.

**Figure 3:**
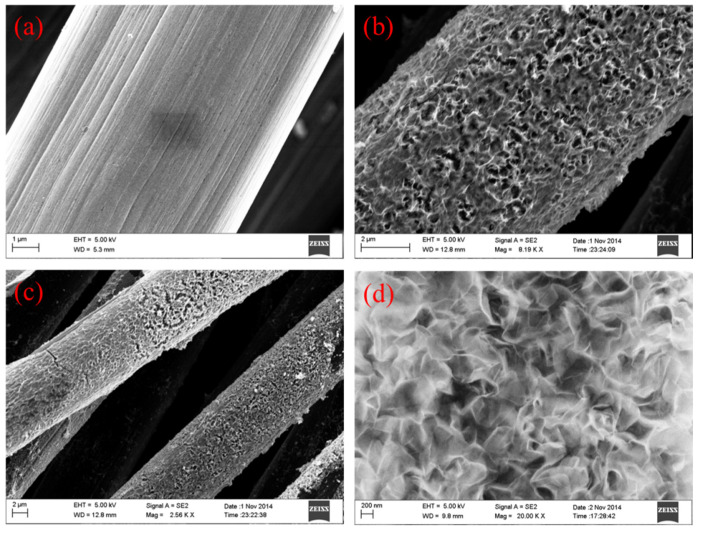
SEM of (**a**) Carbon fibers and (**b**,**c**) NiCo_2_O_4_ Nanosheets@Carbon fibers and (**d**) high resolution SEM images of NiCo_2_O_4_ nanosheets.

SEM characterization was employed to investigate the surface morphology of the NiCo_2_O_4_ NS@CF. In Figure 3a, we could observe that there were carbon fibers of 10 µm diameters with smooth surface. The NiCo_2_O_4_ nanosheets were fabricated on the carbon fibers surface by co-electrodeposition. These NiCo_2_O_4_ nanosheets were aligned vertically on the smooth surface of carbon fibers and crosslinked with each other, producing loosely porous nanostructures (Figure 3b–d). Energy-dispersive X-ray spectroscopy (EDS) was performed to investigate the elements distribution throughout the nanostructure (Figure S6a–c). It revealed that the molar ratio of Ni, Co, and O is about 1:2:4 for the NiCo_2_O_4_ nanosheets@Carbon fibers cloth, which is in good agreement with the stoichiometric ratio of NiCo_2_O_4_. The corresponding EDS mapping images for the elements of Ni, Co, and O illustrate clearly a homogeneous distribution of NiCo_2_O_4_ nanosheets throughout the carbon fibers. TGA curves can exhibit the mass ratio of NiCo_2_O_4_ nanosheets in NiCo_2_O_4_ NS@CF (Figure S10). It is 41.7 wt%. The surface area and the pore size distribution were tested by N_2_ adsorption–desorption isotherms. Type IV isotherms with H4 hysteresis loops indicate the mesoporous structure of NiCo_2_O_4_ NS@CF in Figure S7a. The specific surface area of NiCo_2_O_4_ NS@CF was measured to be 127.1 m^2^ g^−1^. In addition, the pore size distribution of NiCo_2_O_4_ NS@CF exhibits mesopores and micropores. The size of mesopores ranges from 6 to 22 nm (Figure S7b). This feature would create considerable open space and electroactive surface sites for the charge transport and ion diffusion.

**Figure 4:**
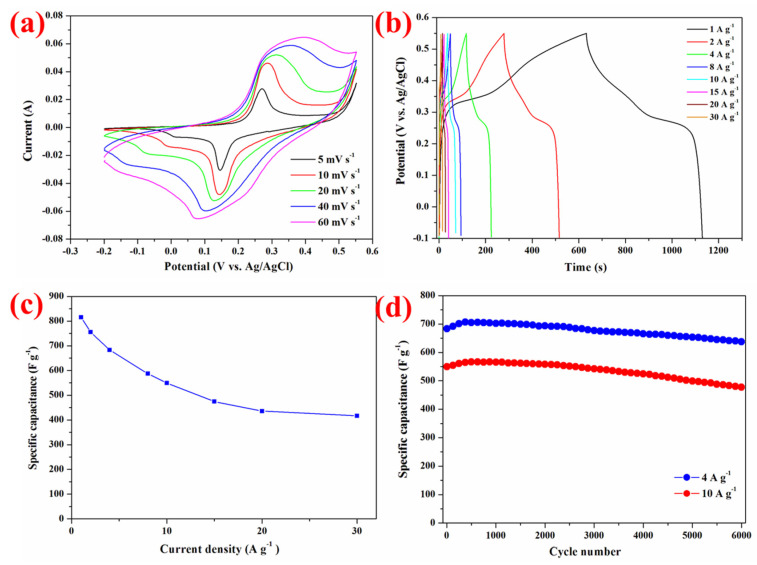
Electrochemical characterizations of the NiCo_2_O_4_ Nanosheets@Carbon fibers cloth. (**a**) Cyclic voltammetry curve at various scan rates from 5 to 60 mV s^−1^. (**b**) Galvanostatic charge–discharge curves at various current densities from 1 to 30 A g^−1^. (**c**) The corresponding specific capacitance as a function of current density. (**d**) Cycling performance at the current density of 4 A g^−1^ and 10 A g^−1^.

The electrochemical performance of the NiCo_2_O_4_ NS@CF was evaluated in a three-electrode cell for supercapacitors. Figure 4a showed its typical cyclic voltammetry (CV) curves at scan rates ranging from 5 to 60 mV s^−1^ in the potential range of −0.20 V to 0.55 V vs. Ag/AgCl. All the CV curves of ultrathin mesoporous NiCo_2_O_4_ nanosheets show well-defined redox peaks within the potential range for all sweep rates. This is attributed to the Faradaic redox reactions related to M-O/M-O-OH (M represents Ni or Co) [26], which manifested the redox pseudocapacitive characteristics of the NiCo_2_O_4_ NS @CF electrode. The current density increased with the increasing sweep rate, revealing the high-power characteristics of the electrode. The redox peaks were observed at 0.15 and 0.29 V at the sweep rate 5 mV s^−1^ while they were at 0.06 to 0.37 V with the sweep rate of 60 mV s^−1^, with a shift of only 0.09 and 0.08 V even by a 12-fold increase in the sweep rate. It suggested low polarization of the electrode due to the good contact between the NiCo_2_O_4_ nanosheets and the carbon fibers cloth electrode, indicating that this hybrid structure is beneficial to fast redox reactions. It is anticipated that the carbon fibers cloth electrode will ensure excellent electrical conductivity within the hybrid structural electrode. This expectation can be corroborated through electrochemical impedance spectroscopy measurements, as depicted in Supporting Information Figure S3.

Galvanostatic charge–discharge behaviors were carried out between −0.1 V and 0.55 V vs. Ag/AgCl at various current densities ranging from 1 to 30 A g^−1^ (Figure 4b). The nearly symmetric shape of all potential–time curves implied the high charge–discharge coulombic efficiency and low polarization of this NiCo_2_O_4_ NS@CF electrode. A well-defined plateau was observed at 0.40 V at all current densities, which agreed well with the CV curves. The specific capacitance was calculated by the formula C_s_ = IΔt/mΔV, where I is the discharge current, Δt is the discharge time, m is the mass of active materials, and ΔV is the voltage range and plotted as a function of the discharge current density. The specific capacitance was as high as 816, 755, 683, 587, 549, 474, 435, and 416 F g^−1^ at the discharge current densities of 1, 2, 4, 8, 10, 15, 20, and 30 A g^−1^ respectively (Figure 4c). Cycling performance was studied with the current density of 4 A g^−1^ and 10 A g^−1^ (Figure 4d). At the current density of 4 A g^−1^, the specific capacitance reached a high value of 683 F g^−1^ in the first cycle and decreased slightly to 637 F g^−1^ after 6000 cycles, with only 6.7% overall capacitance loss. Even at a high discharge current density of 10 A g^−1^, the capacity retention was 87% after 6000 cycles. Based on the overall electrochemical performance, the NiCo_2_O_4_ NS@CF electrode exhibits superior performance compared to many other NiCo_2_O_4_ nanostructured electrodes, as evidenced by the data presented in the Supporting Information Table S1. The enhanced electrochemical performance is supported by the EIS analysis in Figure S3. The ultrathin and mesoporous characteristics of the NiCo_2_O_4_ nanosheets provide numerous electroactive sites for redox reaction. Furthermore, the open space between the ultrathin mesoporous NiCo_2_O_4_ nanosheets structure can serve as a robust reservoir for ions, and enhance the diffusion kinetics. There is direct contact between each NiCo_2_O_4_ nanosheet and carbon fibers substrate, which possesses good intrinsic electrical conductivity and establishes an efficient pathway for electron transport. This design eliminates the need for polymer binders and conductive additives, which typically reduce extra contact resistance.

To demonstrate the excellent capacitive performance of the NiCo_2_O_4_ NS@CF in practical applications, an asymmetric supercapacitor was fabricated using NiCo_2_O_4_ NS@CF and reduced Graphene oxide (rGO) as the positive and negative electrodes, respectively (denoted as NiCo_2_O_4_ NS@CF//rGO). These two electrodes should be balanced by charge to obtain highly effective supercapacitors [27]. Since the charge (q) was calculated by the following equation:

q = C_m_ × m × ΔV (4)

where C_m_ and m are the specific capacitance and mass of the electrode, respectively, and ΔV is the potential window in the charge–discharge process, the mass ratio of NiCo_2_O_4_ NS@CF/rGO is calculated based on the following equation.

m_+_/m_−_ = (C_m−_ × ΔV_−_)/(C_m+_ × ΔV_+_) (5)

**Figure 5:**
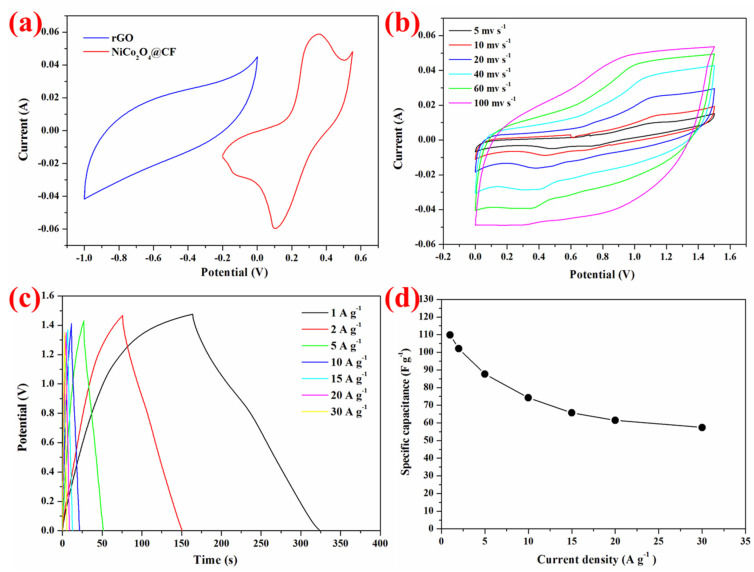
Electrochemical characterizations of the NiCo_2_O_4_ NS@CF//rGO asymmetric supercapacitor. (**a**) Cyclic voltammetry curves of NiCo_2_O_4_ NS@CF and rGO at a scan rate of 60 mV s^−1^ in a three-electrode system. (**b**) CV curves at different scan rates (5 to 100 mV s^−1^). (**c**) Galvanostatic charge–discharge curves at different current densities (1 to 30 A g^−1^). (**d**) Specific capacitance calculated from (**c**) as a function of current density.

The CV curves of NiCo_2_O_4_ NS@CF (red curve) and rGO (blue curve) were measured individually with the potential range of −0.2 V to 0.55 V and −1.0 V to 0 V at a scan rate of 60 mV s^−1^ in Figure 5a, respectively. Both CV curves showed complementary potential windows, indicating the excellent potential of both materials for asymmetric supercapacitor device usage. The CV curves measured at different potential ranges at 100 mV s^−1^ indicated that the potential window can be as large as 1.5 V (Figure S4). Figure S5 presented the galvanostatic charge–discharge curves of the NiCo_2_O_4_ NS@CF//rGO at 1 A g^−1^ with different potential range and showed that the asymmetric supercapacitor device exhibited ideal capacitive properties without an obvious polarization curve even at as large as 1.5 V.

Figure 5b shows the CV curves of the asymmetric supercapacitor device at a scan rate from 5 to 100 mV s^−1^, where all the curves exhibited a similar shape even at a high scan rate of 100 mV s^−1^ and the redox peak currents increased with the increasing scan rate. It indicated that the charge–discharge property of the device was very fast. Moreover, the galvanostatic charge–discharge performance was measured at various current densities from 1 to 30 A g^−1^ in Figure 5c. The discharge curves were nearly linear while the whole galvanostatic charge–discharge curves were triangular in shape, suggesting a rapid I-V response and good electrochemical reversibility of the asymmetric device [28,29]. The specific capacitance of the asymmetric device was calculated based on the total mass of the electrode materials and plotted against current density in Figure 5d. The specific capacitances were 110, 102, 88, 74, 66, 62, and 57 F g^−1^ at 1, 2, 5, 10, 15, 20, and 30 A g^−1^, respectively. Furthermore, 52% of the initial specific capacitance was maintained even with a 30 times increase in current density.

**Figure 6:**
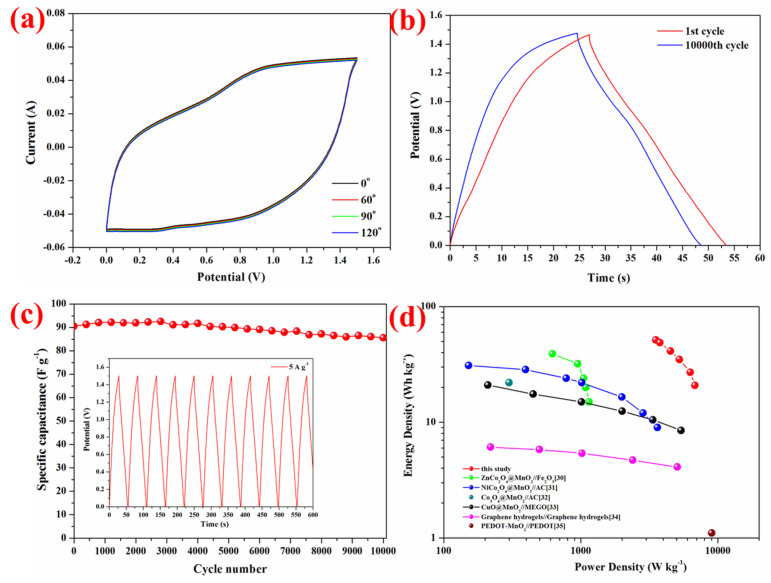
(**a**) CV curves at different bent angles (0° to 120°). (**b**) Galvanostatic charge–discharge curves at 1st and 5000th cycles. (**c**) Cycling performance for 5000 cycles. (**d**) The Ragone plot of NiCo_2_O_4_ NS@CF//rGO asymmetric supercapacitors and other asymmetric supercapacitors reported in the literature.

In order to further demonstrate the practical application of this asymmetric device as a flexible device, the device was mechanically bent and CV curves were measured (Figure 6a). Notably, little changes were observed for the CV curves at different bending angles even up to 120°, suggesting that our NiCo_2_O_4_ NS@CF could be bent to a large extent without decay of its performance.

The cycling stability tests were conducted on the four electrodes at the current density of 5 A/g for 10,000 cycles. The NiCo_2_O_4_ NS@CF//rGO asymmetric device demonstrates excellent cycling stability and maintains a capacitance of 91 F/g with capacitance retention of 94.5% after 10 000 cycles, as illustrated in Figure 6c. And only a slight decrease in charge–discharge time was observed in Figure 6b. The cycling stability is closely associated with the structural stability of the electrodes.

The energy and power densities (E and P) were calculated from the equations $E=\int_{0}^{∆t}IV\left(t\right)dt$ and P=E/∆t, respectively, with I being the discharging current, V being discharging voltage, dt being time differential, and Δt being the discharge time. Regone plots of the NiCo_2_O_4_ NS@CF//rGO asymmetric device and other reported materials were shown in Figure 6d to further evaluate the performance. The NiCo_2_O_4_ NS@CF//rGO asymmetric device delivered a high energy density of 52 Wh kg^−1^ with a power density of 3.5 kW kg^−1^ and high power density of 7.1 kW kg^−1^ with an energy density of 21 Wh kg^−1^, which were higher than those of most nickel and cobalt-based asymmetric supercapacitors including ZnCo_2_O_4_@MnO_2_//Fe_2_O_3_ [30], NiCo_2_O_4_@MnO_2_//AC [31], and Co_3_O_4_@MnO_2_//AC [32]; copper-based asymmetric supercapacitors CuO@MnO_2_//MEGO [33]; and symmetric supercapacitor Graphene hydrogels//Graphene hydrogels [34]. Although PEDOT-MnO_2_//PEDOT can achieve slightly higher power density [35], their energy density was at least 20 times lower. These results indicated that NiCo_2_O_4_ NS@CF//rGO is a promising candidate as an asymmetric supercapacitor device.

## 4. Conclusions

In summary, we have proposed the general guidelines for designing high-performance supercapacitor electrodes and illustrated these principles through practical demonstrations. This involved combining NiCo_2_O_4_ nanosheets with carbon fibers cloth, resulting in a core-shell nanostructure. The significance of our electrode lies in several key aspects. Firstly, we utilize lightweight and highly conductive carbon fibers as the integrated electrode substrate. Secondly, the incorporation of ultrathin mesoporous NiCo_2_O_4_ nanosheets in a homogeneous manner enhances rate capability and structural stability. Through comprehensive comparative experiments, we have demonstrated that asymmetric supercapacitors employing these highly integrated electrodes exhibit superior electrochemical properties, including higher specific capacitance, high energy density, high power density, and improved cycling stability. It is envisaged that the unique nano-architecture of this hybrid electrode may be applicable to other chemical and energy transformation processes such as lithium ion batteries, water splitting, photo-detector, and non-enzymatic biosensors.

## Data Availability

The data presented in this study are available on request from the corresponding author.

## Supplementary Materials

The following supporting information can be downloaded at: https://www.mdpi.com/article/10.3390/nano15010029/s1. Figure S1: SEM image of carbon fibers cloth; Figure S2: Specific capacitance of NiCo_2_O_4_ nanosheets on carbon fibers cloth based on different electrodeposition time; Figure S3: Nyqusit plot of the NiCo_2_O_4_ nanosheets@carbon fibers electrode. Nyqusit plot of the NiCo_2_O_4_ NS@CF, NiCo_2_O_4_ NS@stainless steel and NiCo_2_O_4_+ conductive agent+binder@CF; Figure S4: CV curves of the asymmetric supercapacitor devices at different potential windows with the scan rate of 100 mV s^−1^; Figure S5: Galvanostatic charge–discharge curves at different potential windows with the current density of 1 A g^−1^; Figure S7: (a) N2 adsorption and desorption isotherm of NiCo_2_O_4_ NS@CF. (b) Pore size distribution of NiCo_2_O_4_ NS@CF; Figure S8: X-ray diffraction pattern of Carbon fibers cloth; Figure S9: X-ray diffraction pattern of NiCo_2_O_4_ Nanosheets @Carbon fibers; Figure S10: TGA curve of of NiCo_2_O_4_ Nanosheets @Carbon fibers in air; Table S1: Electrochemical performance of the NiCo_2_O_4_ nanosheets@carbon fibers in this study, compared with some other NiCo_2_O_4_ electrodes reported in previous literature. References [36,37,38,39,40,41] are cited in the Supplementary Materials.
