# Supplementary material for: Facile Synthesis of Mesoporous NiCo2O4 Nanosheets on Carbon Fibers Cloth as Advanced Electrodes for Asymmetric Supercapacitors
Source: Nanomaterials (Basel). 2024 Dec 27;15(1):29. doi: 10.3390/nano15010029 (PMC11722020; doi:10.3390/nano15010029)
Supplement: Supplementary file 1 [file nanomaterials-15-00029-s001.zip › nanomaterials-2962891-supplementary.pdf]

nanomaterials

Supplementary Materials

Facile Synthesis of Mesoporous $NiCo_2O_4$ Nanosheets on Carbon Fibers Cloth as Advanced Electrodes for Asymmetric supercapacitors

Xiang Zhang

Department of Energy and Power Engineering, North University of China, Taiyuan 038507, China

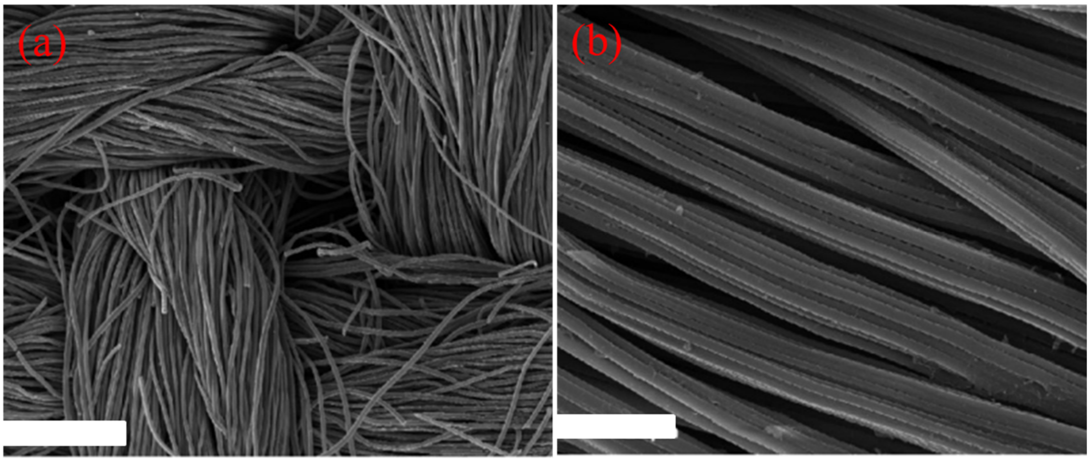

Figure S1. SEM image of carbon fibers cloth.

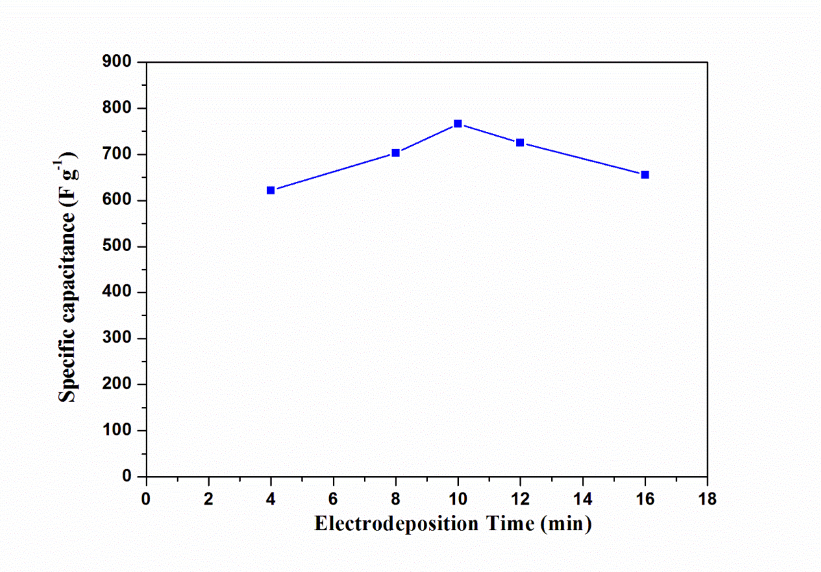

Figure S2. Specific capacitance of $NiCo_2O_4$ nanosheets on carbon fibers cloth based on different electrodeposition time.

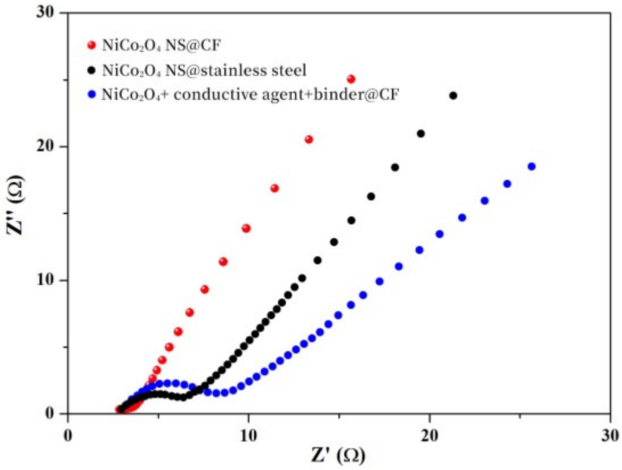

Figure S3. Nyqusit plot of the $NiCo_2O_4$ nanosheets@carbon fibers electrode. Nyqusit plot of the $NiCo_2O_4$ NS@CF, $NiCo_2O_4$ NS@stainless steel and $NiCo_2O_4$+ conductive agent+binder@CF

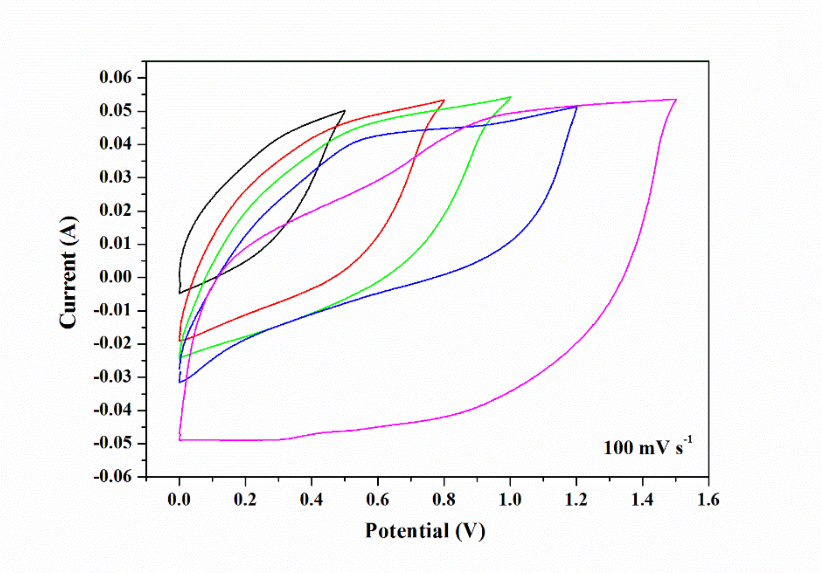

Figure S4. CV curves of the asymmetric supercapacitor devices at different potential windows with the scan rate of 100 mV s^{-1}.

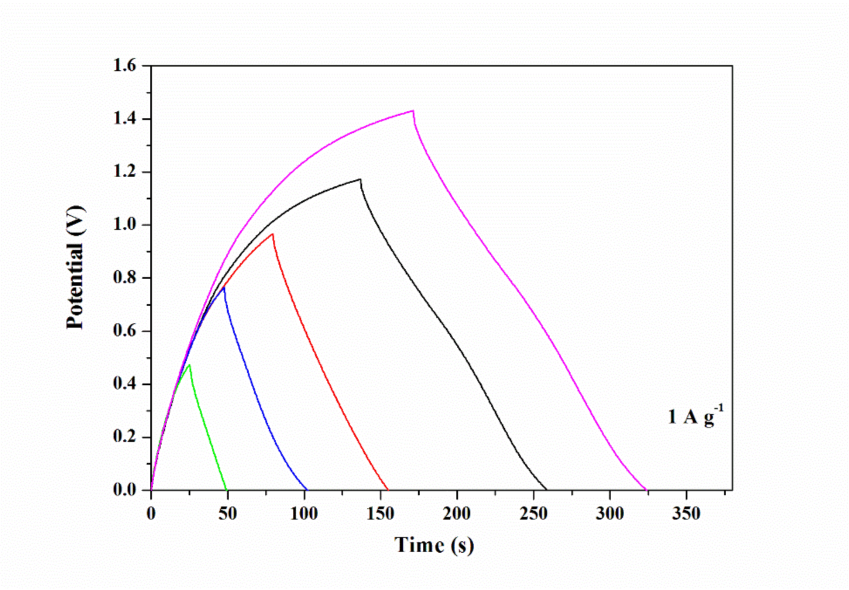

Figure S5. Galvanostatic charge–discharge curves at different potential windows with the current density of 1 A $g^{-1.}$

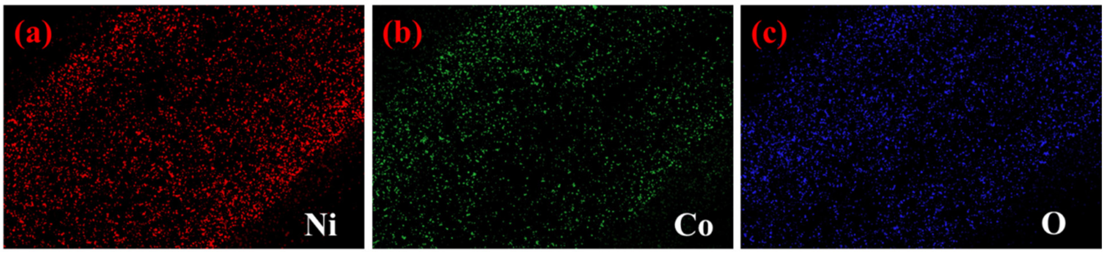

Figure S6. SEM image of $NiCo_2O_4$ nanosheets @Carbon fibers cloth with elements mapping, (a)–(c) Element mappings of Co (red), Ni (blue) and oxygen (blue), respectively.

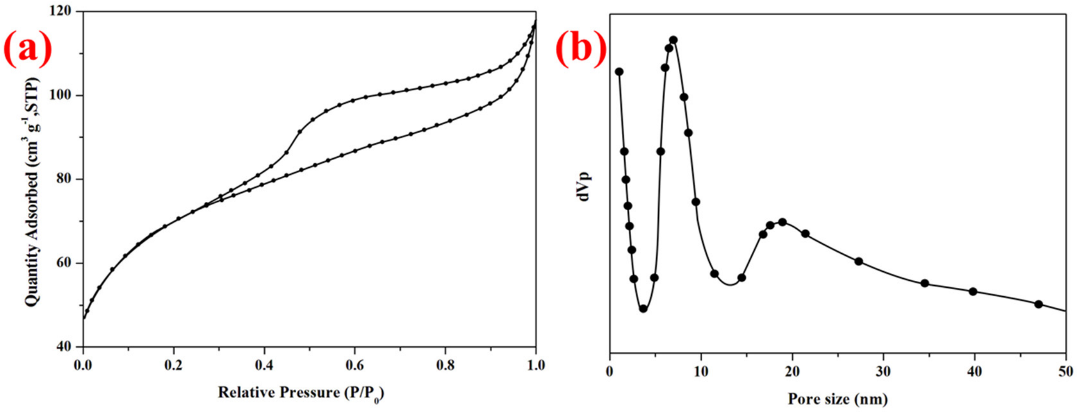

Figure S7. (a) N_2 adsorption and desorption isotherm of $NiCo_2O_4$ NS@CF. (b) Pore size distribution of $NiCo_2O_4$ NS@CF

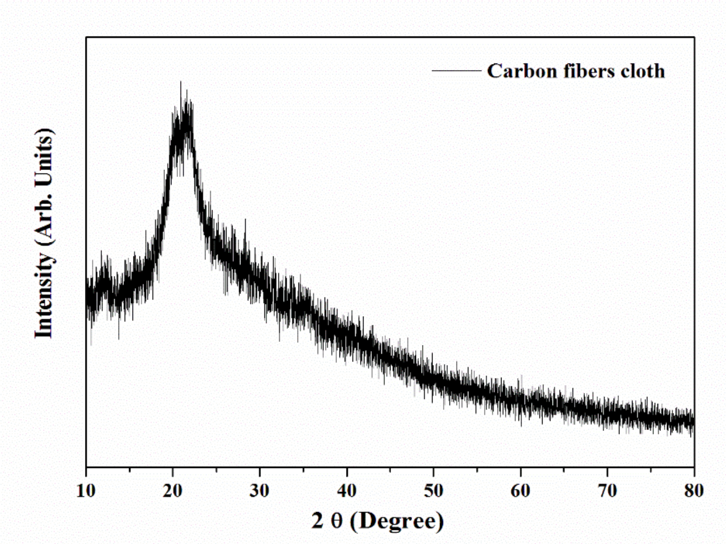

Figure S8. X-ray diffraction pattern of Carbon fibers cloth

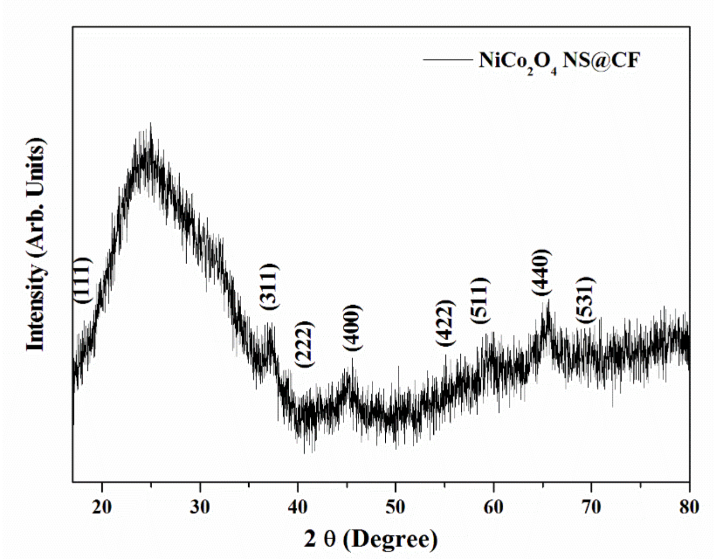

Figure S9. X-ray diffraction pattern of $NiCo_2O_4$ Nanosheets @Carbon fibers

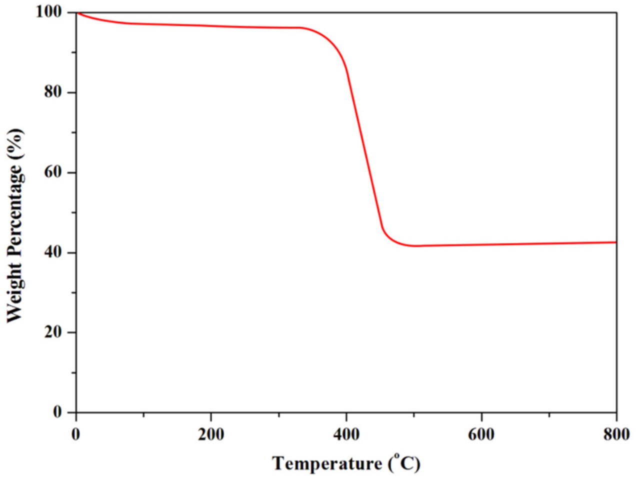

Figure S10. TGA curve of of $NiCo_2O_4$ Nanosheets @Carbon fibers in air

TGA curves can exhibits the mass ratio of $NiCo_2O_4$ nanosheets in $NiCo_2O_4$ NS@CF. The weight of the electrode active materials was calculated by the follows: weight of $NiCo_2O_4$ NS@CF × the mass ratio of $NiCo_2O_4$ nanosheets, the mass loading of $NiCo_2O_4$ nanosheets on carbon fibers was calculated by the following equation:

Weight of $NiCo_2O_4$ NS@CF × the mass ratio of $NiCo_2O_4$ nanosheets) / the area of $NiCo_2O_4$ NS@CF.

Table S1. Electrochemical performance of the $NiCo_2O_4$ nanosheets@carbon fibers in this study, compared with some other $NiCo_2O_4$ electrodes reported in previous literature.

**Materials**	**Specific Capacity (F g^{-1})**	**Capacity Degradation after Cycling**	**Capacity Retention**	**Ref.**
$NiCo_2O_4$ NS@CF	549 at 10 A g^{-1}	6.9% after 3000 cycles; 13% after 6000 cycles	67.3% from 1 to 10 A g^{-1}	This Study
Urchin-like $NiCo_2O_4$	530 at 10 A g^{-1}	2% after 1000 cycles	81% from 1 to 10 A g^{-1}	1
$NiCo_2O_4$ nanowire	532 at 20 A g^{-1}	19% after 3000 cycles	70% from 1 to 20 A g^{-1}	2
$NiCo_2O_4$ crystals	188 F g^{-1} at 20 mA cm^{-2}	4% after 600 cycles	87% from 1 to 20 mA cm^{-2}	3
$NiCo_2O_4$ nanoparticles	349 F g^{-1} at 16 A g^{-1}	52% after 300 cycles	53% from 1 to 16 A g^{-1}	4
$NiCo_2O_4$ nanosheets	202 F g^{-1} at 10 A g^{-1}	6% after 2000 cycles	40% from 1 to 10 A g^{-1}	5
Ultrathin Mesoporous $NiCo_2O_4$ Nanosheets@Ni foam	2010 F g^{-1} at 2 A g^{-1}	No degradation after 400 cycles	72.1% from 1 to 20 A g^{-1}	6
$Ni(OH)_2$@$NiCo_2O_4$ Grown on Carbon Fiber Paper	5.2 F cm^{-1} at 2 mA cm^{-2}	64% after 1000 cycles	79% from 2 to 50 mA cm^{-2}	7
Nickel–Cobalt Hydroxide Nanosheets Coated on $NiCo_2O_4$ Nanowires@Carbon Fiber Paper	2.3 F cm^{-1} at 2 mA cm^{-2}	28% after 2000 cycles	60.8% from 2 to 150 mA cm^{-2}	8

References

1. Y. Zhang, B. Wang, F. Liu, J. Cheng, X.-w. Zhang and L. Zhang, *Nano Energy*, 2016, **27**, 627-637.
2. H. Wang, Q. Gao and L. Jiang, *Small*, 2011, **7**, 2454-2459.
3. Y. Q. Wu, X. Y. Chen, P. T. Ji and Q. Q. Zhou, *Electrochimica Acta*, 2011, **56**, 7517-7522.
4. J. Xiao and S. Yang, *RSC Advances*, 2011, **1**, 588-595.
5. X. Lu, X. Huang, S. Xie, T. Zhai, C. Wang, P. Zhang, M. Yu, W. Li, C. Liang and Y. Tong, *Journal of Materials Chemistry*, 2012, **22**, 13357-13364.
6. C. Yuan, J. Li, L. Hou, X. Zhang, L. Shen and X. W. Lou, *Advanced Functional Materials*, 2012, **22**, 4592-4597.
7. L. Huang, D. Chen, Y. Ding, Z. L. Wang, Z. Zeng and M. Liu, *ACS Applied Materials & Interfaces*, 2013, **5**, 11159-11162.
8. L. Huang, D. Chen, Y. Ding, S. Feng, Z. L. Wang and M. Liu, *Nano Letters*, 2013, **13**, 3135-3139.
